# The use of transcutaneous bilirubin nomograms for the prevention of bilirubin neurotoxicity in the neonates

**DOI:** 10.3389/fpubh.2023.1212667

**Published:** 2023-07-19

**Authors:** Lucia Casnocha Lucanova, Jana Zibolenova, Katarina Matasova, Katarina Matasova, Mirko Zibolen

**Affiliations:** ^1^Neonatology Department, Jessenius Faculty of Medicine in Martin, Comenius University in Bratislava, University Hospital Martin, Martin, Slovakia; ^2^Department of Public Health, Jessenius Faculty of Medicine in Martin, Comenius University in Bratislava, Martin, Slovakia

**Keywords:** bilirubin, jaundice, prevention of hyperbilirubinemia, neonate, nomogram, transcutaneous bilirubinometry

## Abstract

**Purpose:**

Although neonatal jaundice is a ubiquitous and predominantly benign phenomenon, the risk of neurotoxicity exists in a number of infants with unconjugated hyperbilirubinemia. Plotting bilirubin values on nomograms enables clinicians to employ an anticipatory and individualized approach with the goal of avoiding excessive hyperbilirubinemia and preventing acute bilirubin encephalopathy and its progression to kernicterus. We aimed to construct nomograms for White term infants based on transcutaneous bilirubin (TcB) measurements using a JM-105 device.

**Methods:**

TcB measurements were taken in infants at ages ranging from 0 to 96 postnatal hours. We then constructed hour-specific TcB nomograms from forehead and sternum measurements in infants who did not require subsequent phototherapy.

**Results:**

We included 2,981 TcB measurements taken on the forehead and 2,977 measurements taken on the sternum in 301 White term newborn infants. We assessed the predictive abilities of the nomograms at six postnatal time intervals using receiver operating characteristic curves. The areas under the curves indicated reasonable prediction of hyperbilirubinemia requiring phototherapy, except for the forehead measurement taken within the first 12 h of life. Sensitivity tended to rise as postnatal age increased.

**Conclusion:**

The nomograms illustrate dermal bilirubin dynamics in White term neonates during the first 4 days of life. They may be useful tools to predict individualized risk of hyperbilirubinemia requiring treatment, and to plan optimal follow-up of infants at risk of bilirubin neurotoxicity.

## Introduction

Neonatal unconjugated hyperbilirubinemia and resultant clinical jaundice affect approximately 85% of neonates. Although generally a benign, transitional phenomenon, excessive unconjugated bilirubin levels can pose a direct threat of serious brain injury in a small proportion of neonates (1). Acute bilirubin encephalopathy (ABE) remains a significant cause of neonatal morbidity and mortality (2). An estimated 1.1 million newborn infants worldwide develop total serum bilirubin (TSB) >20 mg/dL. There were 114,000 neonatal deaths associated with extreme neonatal hyperbilirubinemia (TSB >25 mg/dL) and Rh disease in 2010 (3). Kernicterus is typically characterized by choreoathetoid cerebral palsy, sensorineural hearing loss, paralysis of upward gaze, and dental enamel dysplasia. The estimated incidence of kernicterus ranges from 0.4 to 2.7 cases per 100,000 live births among term and late preterm neonates in North America and Europe (4). A re-emergence of kernicterus has been reported following a shift to early hospital discharge after delivery in high-income countries (5). Moreover, bilirubin-induced neurological disorders (BIND) caused by exposure to high levels of bilirubin can occur in the absence of classical kernicterus, leading to disturbances in visuo-oculomotor, auditory, speech, cognition, and language processes (6).

Timely recognition of rapidly rising TSB levels or severe hyperbilirubinemia (TSB >20 mg/dL) is crucial to avoid bilirubin neurotoxicity. Visual assessment of jaundice is not reliable. Objective screening methods such as routinely available noninvasive transcutaneous bilirubinometry can facilitate early recognition and timely treatment of hyperbilirubinemia (7). The American Academy of Pediatrics (AAP) acknowledges that TSB levels can be estimated by using noninvasive transcutaneous bilirubin (TcB) measurements. TcB or TSB should be measured between 24 and 48 h after birth or before discharge if that occurs earlier according to the revised 2022 AAP guideline (8). The purpose of TcB measurement is to identify when to sample the blood for TSB assessment. However, it is important not to rely on a single TcB value, but rather to recognize the dynamics of bilirubin concentrations.

TcB nomograms illustrate the natural history of TcB accumulation in specific populations of newborn infants. Hour-specific bilirubin nomograms are useful to detect aberrant trends, to identify infants in need of further evaluation, and to plan appropriate follow-up care (9). We aimed to construct dermal bilirubin nomograms for White term infants using a JM-105 device. Plotting TcB values on such nomograms may enable clinicians to employ an anticipatory and individualized approach with the goal of avoiding severe hyperbilirubinemia. The predictive and personalized strategy is key to prevent severe neonatal hyperbilirubinemia, ABE, and its subsequent progression to kernicterus.

## Materials and methods

This prospective non-interventional study was approved by the Ethical Committee of Jessenius Faculty of Medicine in Martin, Comenius University Bratislava (IRB00005636), registered by the Office for Human Research Protection, U.S. Department of Health and Human Services (IORG0004721). The ethical committee registration number is EK35/2021. Parental informed consent was obtained from all subjects.

Healthy term newborn infants born at University Hospital Martin were enrolled in the study. The inclusion criteria were White race (both parents are White), gestational age ≥ 36 weeks, and Apgar score ≥ 7 at 5 min of life. The exclusion criteria included cephalohematoma, significant bruising, Apgar score < 7 at 5 min of life, suspicion of hemoglobinopathy, disorders of the erythrocyte membrane, liver diseases, cholestasis, delayed passage of meconium (failure to pass meconium within 24 h of life), hypoglycemia, hypothermia (rectal temperature < 36.5°C), hypothyroidism, major congenital malformations, neonatal hemorrhagic disease, clinical diagnosis of sepsis or perinatal infection, respiratory distress syndrome, and pharmacological treatment except for vitamin K administered intramuscularly immediately after birth. TcB concentrations were not measured in newborns during and after phototherapy or after exchange transfusion. TcB measurements were not made on any anomalous part of the skin such as birthmarks, nevi, or hairy areas.

TcB values were obtained with a JM-105 Jaundice Meter, which determines the yellowness of the subcutaneous tissue by measuring the difference in the optical densities for light in the blue (450 nm) and green (550 nm) wavelength regions. Because the optical density difference shows a linear correlation with the TSB concentration, it is converted to the estimated bilirubin concentration and displayed digitally (10). The TcB value was determined as a calculated mean value of three consecutive readings for each measurement site: the forehead (above the glabella) and the sternum (mid-sternum). A single JM-105 device was used and calibrated regularly.

TcB measurements were performed at designated times from 0 to 96 h of postnatal age. The time interval between two TcB measurements in a single infant was approximately 6 h; postnatal age was recorded for every TcB measurement. Blood sampling for TSB measurements was performed based on clinical indications, TcB values and their trend in a specific patient, risk factors for developing severe hyperbilirubinemia, and postnatal age (8). Phototherapy was initiated according to AAP recommendations (11, 12).

## Statistical analysis

Newborns were divided into two groups according to their need for phototherapy. Continuous variables (gestational age and birth weight) were categorized, and all categorical variables (gender, delivery mode, feeding, and risk factors) are presented as absolute numbers and relative frequencies. The association between categorical variables and the need for subsequent phototherapy was analyzed by using the chi-square test with Yates correction.

TcB nomograms for the forehead and sternum measurement sites were constructed for neonates without phototherapy. For the group of infants in whom phototherapy was subsequently indicated, 50th percentile curves were constructed. TcB percentile curves (5th, 10th, 25th, 50th, 75th, 90th, and 95th) depending on postnatal age were obtained using quantile regression in R (the quantreg package: polynomial fit) (13, 14). Plots and rates of TcB increase were constructed using Microsoft Excel 2019 (Microsoft, Redmond, WA, United States).

Receiver operating characteristic (ROC) curves were used to determine the predictive abilities of the nomograms for the subsequent need of phototherapy. The discrimination thresholds were determined as maximum TcB percentiles in the appropriate 12-h interval according to the constructed nomograms. Areas under the curves (AUC) and 95% confidence intervals (95% CI) were calculated using the ROCR package (15) and SPSS Statistics for Windows Version 29.0 (IBM Corp., Armonk, NY, USA). Sensitivity, and specificity, negative predictive values, and positive predictive values for the 95th, 75th, and 40th percentile cut-offs were calculated for each of the six postnatal 12-h intervals.

## Results

The study group consisted of 301 term newborn White infants (141 females and, 160 males). The demographic and clinical data are presented in Table 1, according to the need for subsequent phototherapy. Hyperbilirubinemia that required phototherapy was documented in 25 (8.3%) infants. Overall, 2,981 measurements were performed on the forehead and 2,977 measurements were performed on the sternum. There was a mean of 10 TcB measurements per patient. The need for subsequent phototherapy was more frequent in neonates at a lower gestational age; vaginal delivery; and with known risk factors such as blood group incompatibility, cephalohematoma, and petechiae on the face.

**Table 1:** Study group baseline characteristics according to phototherapy indication (*n* = 301).

Characteristic	No phototherapy	Phototherapy	*p* value
	*n* (%)	*n* (%)	
Study sample	276 (91.7%)	25 (8.3%)	
Number of TcB measurements			
Forehead	2,788 (93.5%)	193 (6.5%)	
Sternum	2,784 (93.5%)	193 (6.5%)	
Gender			
Female	130 (47.1%)	11 (44%)	0.7660
Male	146 (52.9%)	14 (56%)	
Gestational age			
36 + 37	30 (10.9%)	8 (32%)	0.0063
38+	246 (89.1%)	17 (68%)	
Delivery mode			
Vaginal	179 (64.9%)	23 (92%)	0.0131
Cesarean	96 (34.8%)	1 (4%)	
Instrumental	1 (0.4%)	1 (4%)	
Feeding			
Breastfed	45 (16.3%)	2 (8%)	0.4924
Formula	2 (0.7%)	0 (0%)	
Combined	229 (83%)	23 (92%)	
Birth weight (g)			
Mean ± standard deviation	3,423 ± 460	3,358 ± 449	0.644 (*t*-test)
Nutritional status/ Intrauterine growth			
Eutrophy/Appropriate for gestational age	246 (89.1%)	22 (88%)	0.9057
Hypotrophy/Small for gestational age	12 (4.3%)	1 (4%)	
Hypertrophy/Large for gestational age	18 (6.5%)	2 (8%)	
Risk factors			
Blood group incompatibility	11 (4%)	6 (24%)	<0.0001
Cephalohematoma	9 (3.3%)	5 (20%)	0.0009
Petechiae on the face (due to congestion)	31 (11.2%)	8 (32%)	0.0081

TcB, transcutaneous bilirubin.

**Figure 1:**
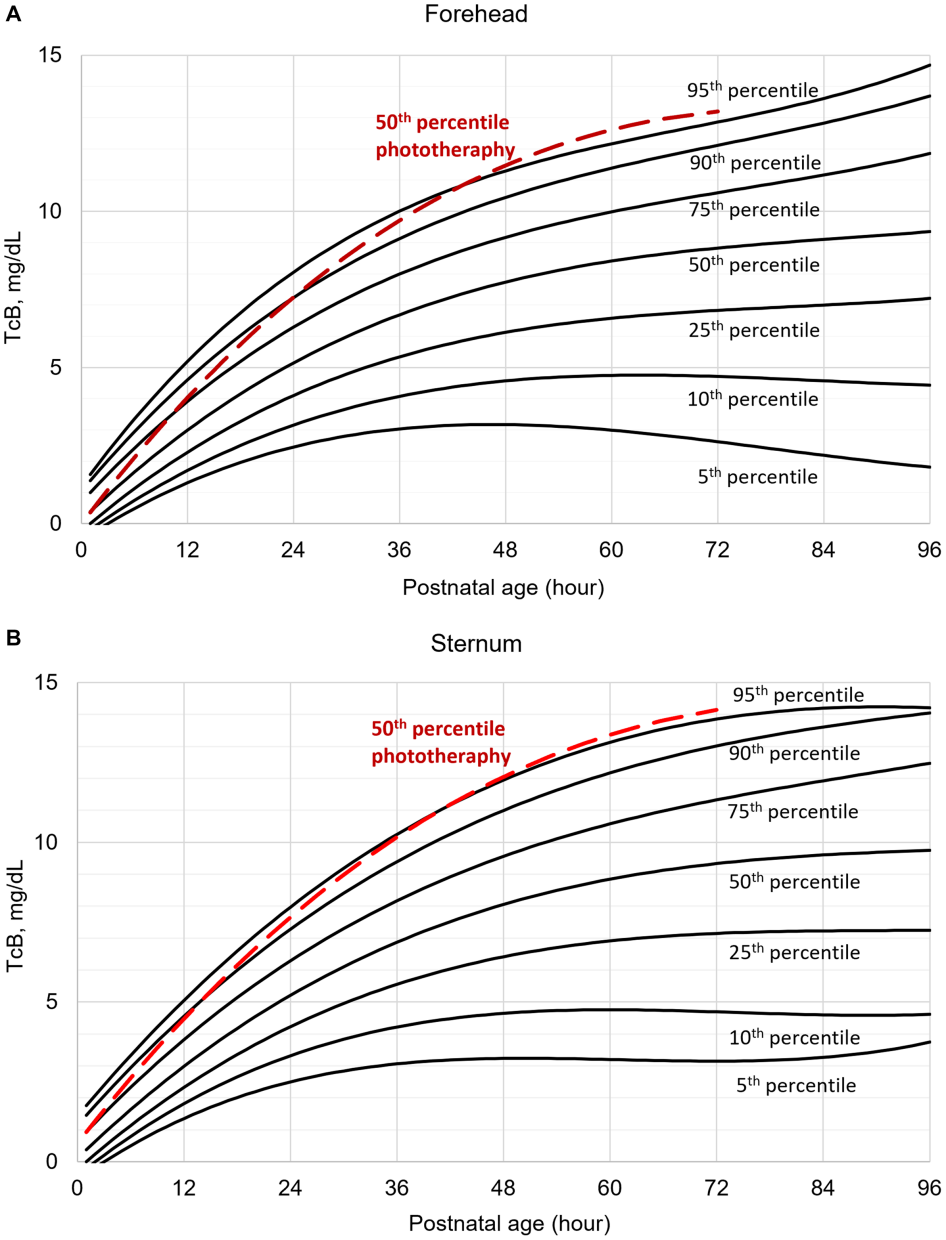
Transcutaneous bilirubin (TcB) nomogram based on measurements on the: **(A)** forehead, **(B)** sternum. The red curve represents the 50th percentile for individuals who required subsequent phototherapy.

Individual TcB measurements according to postnatal age are shown graphically for the forehead and sternum measurement sites (Supplementary Figure 1). The values obtained from infants with subsequent phototherapy are highlighted. TcB nomograms with percentile curves for the forehead and sternum measurement sites are shown in Figure 1. The 50th percentile curves for infants with subsequent phototherapy are also shown. These curves gradually intersect all displayed percentile curves indicating that sharp increases in TcB values predict the need for subsequent phototherapy. The rates of increases of the 50th TcB percentile in neonates with subsequent phototherapy are substantially higher than in neonates without subsequent phototherapy (Supplementary Table 1).

**Figure 2:**
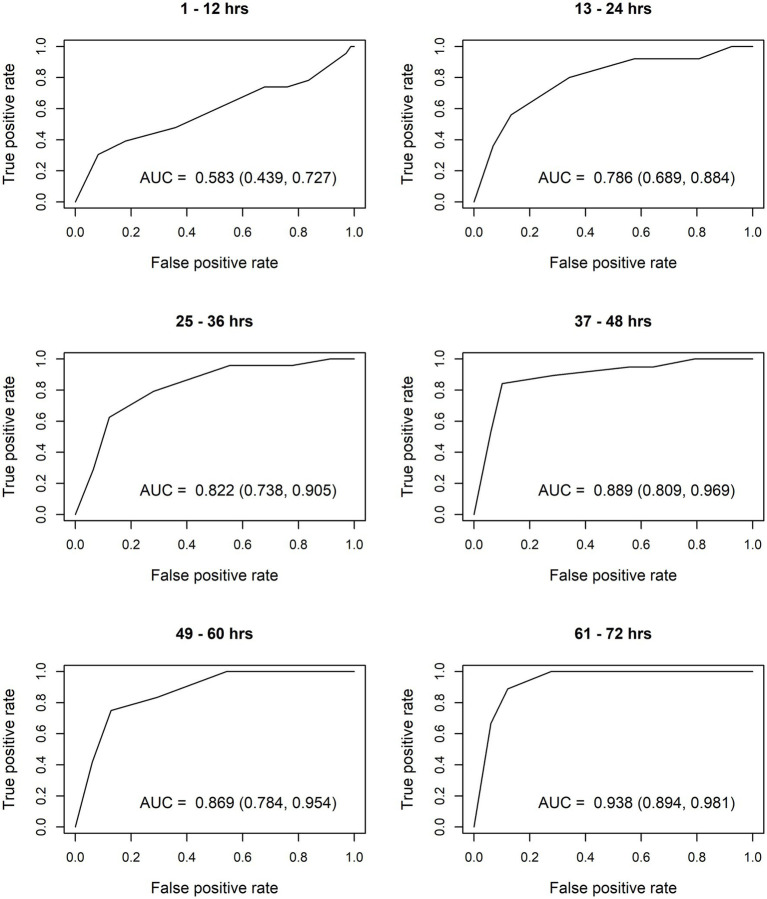
Receiver operating characteristic (ROC) curves based on transcutaneous bilirubin (TcB) measurements taken on the forehead. AUC, area under curve.

**Figure 3:**
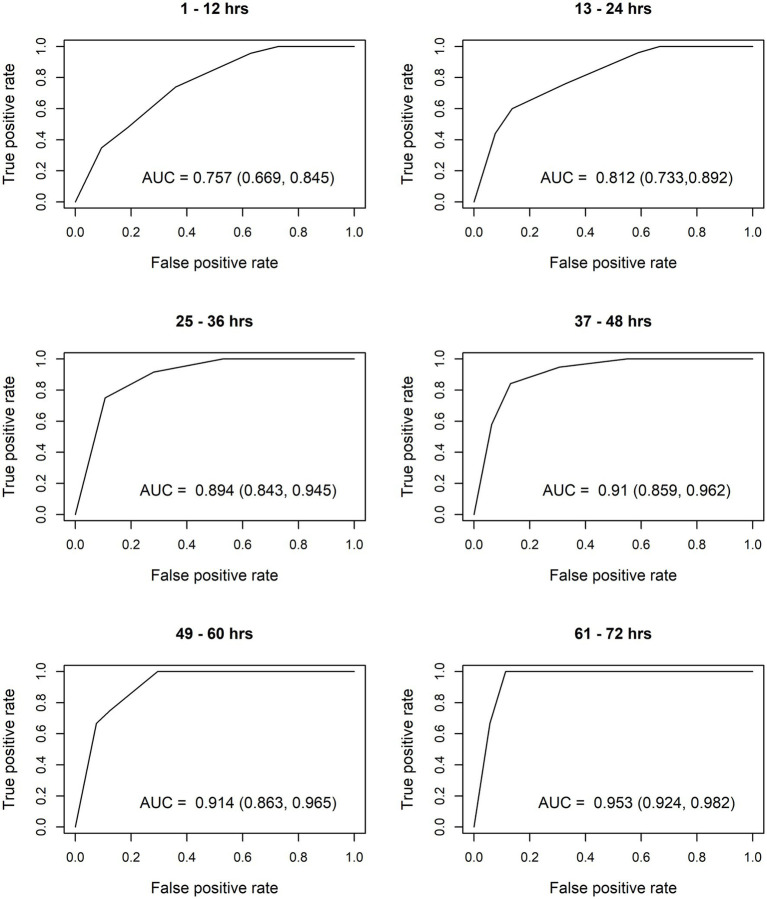
Receiver operating characteristic (ROC) curves based on transcutaneous bilirubin (TcB) measurements taken on the sternum. AUC, area under curve.

After constructing the nomograms, we assessed their predictive abilities for six postnatal intervals (Figures 2, 3). In general, the predictive ability of the subsequent need of phototherapy is better for the sternum measurement site compared with the forehead, and it becomes more accurate as the postnatal age increases. All AUC are significant, except for the forehead measurements taken within the first 12 h of life. More information about the predictive abilities of the constructed nomograms is shown in Supplementary Table 2 for the forehead measurement site, and in Supplementary Table 3 for the sternum measurement site. We found that sensitivity tends to rise as the postnatal age increases within each highlighted cut-off level.

## Discussion

Although unconjugated bilirubin has recently been recognized as one of the most potent endogenous antioxidant substances (16), bilirubin-induced brain damage continues to be an important sequela among newborns worldwide (1). Recent data have documented that neonatal hyperbilirubinemia causes significant, long-lasting negative effects on the developing visual cortex, leading to visuocortical dysfunction even at TSB levels not considered harmful (17). Individual prediction, targeted prevention, and effective personalized treatment of hyperbilirubinemia may play a major role in eliminating long-term neurologic sequelae found in neonates with ABE.

We believe that noninvasive screening is optimal in developing the best possible bilirubin management approach. It is important to avoid unnecessary blood draws in neonatal care. Painful stimuli due to frequent blood sampling can impact the developing neonatal brain, leading to significant adverse long-term neurodevelopmental outcomes (18). Transcutaneous bilirubinometry is painless, user friendly, and easy to learn, and it provides immediate results. We have shown in our previous studies that TcB measurements taken on the forehead and sternum are reliable in White term neonates not treated with phototherapy (19, 20). There is a close and significant correlation between TcB and TSB concentrations in infants with TSB not exceeding 15 mg/dL (8).

The rate at which TSB values rise is useful to identify neonates at risk of severe hyperbilirubinemia (7). Bhutani et al. (21) developed the first hour-specific TSB nomogram in 1999; the limitation in their study was the fact that they only collected data from newborns who had at least one outpatient follow-up TSB. Such a nomogram does not illustrate the natural history of neonatal bilirubin. Moreover, plotting TcB values on TSB nomograms, which is routinely done by clinicians, may be inappropriate and inaccurate to predict the risk of significant hyperbilirubinemia (22). The first TcB nomogram was constructed in the United States in 2006 (9). The study sample consisted of a racially diverse group of infants, and TcB measurements were obtained using the older JM-103 device. The authors highlighted that normal values are needed to allow appropriate interpretation of TcB measurements (9). TcB values should be plotted on nomograms derived from the neonate’s racial and ethnic group (23).

The magnitude of the difference between TcB and TSB concentrations depends on the skin melanin concentration and on the instrument used for TcB measurements (8). The impact of race or skin color on the TcB measurements has been well documented. Moreover, considering normal TSB progression, Black neonates have lower initial TSB concentration values and Asian neonates have higher initial TSB values (21). There are only two TcB nomogram studies based on measurements in European term neonates using BiliChek devices, which incorporate multiple wavelengths in the readings (24, 25). On the other hand, JM devices use a different measurement principle based on two optical paths. We speculate that Italian and Greek infants may differ in the degree of skin melanin concentrations compared with Central European neonates. Discrepancies in bilirubin metabolism have been reported among the various ethnic groups (22). Researchers have suggested that TcB results should be evaluated on a TcB nomogram constructed from infants of a similar racial background and using a similar TcB device (23, 24). This fact highlights the necessity of using a nomogram constructed from European term infants based on JM-105 determinations. Therefore, we aimed to describe the dynamics of dermal bilirubin accumulation measured with the JM-105 device on two different body sites, namely the forehead and sternum.

Our data illustrate normal TcB values in the initial four postnatal days in White term newborn infants. TcB values increase gradually during the first three postnatal days. As expected, TcB increases rapidly in the first 36 h of life. The rates of rise decrease later, and TcB values stabilize approximately at the 60th postnatal hour. The results (Figure 1) document that TcB values exceeding the 75th percentile during the first day of life, and TcB values exceeding the 90th percentile during the second day of life are associated with a high incidence of significant hyperbilirubinemia requiring subsequent phototherapy.

The calculated AUC confirm good predictive ability for subsequent need of phototherapy, with the sternum being the superior measurement site. TcB measurements become more accurate with increasing postnatal age. After the first 12 h of life, TcB is reliable to predict serious hyperbilirubinemia in White term neonates.

Our study has some limitations. It represents results from a single center. The total number of infants for the referred postnatal periods differ for two reasons. First, TcB was not measured at each time in all infants. Second, we excluded infants requiring subsequent phototherapy from the study at the moment the phototherapy started. Consequently, the predictive abilities of the nomograms in later postnatal periods could be influenced by the low number of infants requiring subsequent phototherapy.

There are several strengths of our study. First, the study population is racially and ethnically homogenous, as all neonates are White. Second, all TcB measurements were done routinely, and not because the infant was jaundiced. A quite robust number of TcB measurements (2,981 measurements on the forehead and 2,977 on the sternum) and the inclusion and exclusion criteria allow us to conclude that the presented nomograms illustrate the natural history of dermal bilirubin accumulation in White term newborns. The constructed nomograms should be validated to verify their reliability to predict serious hyperbilirubinemia.

Normal values of TcB are necessary for appropriate interpretation of results of noninvasive screening for significant hyperbilirubinemia. Indeed, clinical judgment and attention to risk factors must be incorporated when using population-specific nomograms.

## Conclusion

We constructed hour-specific TcB nomograms based on measurements on the forehead and sternum of White term neonates. These nomograms document the dynamics of bilirubin accumulation during the first 4 days of life.

TcB nomograms can help identify and predict infants at high risk of developing severe hyperbilirubinemia, indicate timely treatment of significant hyperbilirubinemia, and plan optimal follow-up of infants at risk of bilirubin neurotoxicity.

## Data availability statement

The raw data supporting the conclusions of this article will be made available by the authors, without undue reservation.

## Ethics statement

The studies involving human participants were reviewed and approved by Ethical Committee of Jessenius Faculty of Medicine in Martin, Comenius University Bratislava (IRB00005636), registered by the Office for Human Research Protection, U.S. Department of Health and Human Services (IORG0004721). The ethical committee registration number is EK35/2021. Written informed consent to participate in this study was provided by the participants’ legal guardian/next of kin.

## Author contributions

LCL and KM Jr. contributed to material preparation and data collection. JZ completed data analysis. LCL and JZ drafted the manuscript. All authors commented on previous versions of the manuscript, contributed to the study conception and design, and read and approved the final manuscript.

## Conflict of interest

The authors declare that the research was conducted in the absence of any commercial or financial relationships that could be construed as a potential conflict of interest.

## Publisher’s note

All claims expressed in this article are solely those of the authors and do not necessarily represent those of their affiliated organizations, or those of the publisher, the editors and the reviewers. Any product that may be evaluated in this article, or claim that may be made by its manufacturer, is not guaranteed or endorsed by the publisher.

## Abbreviations

AAP, The American Academy of Pediatrics
ABE, acute bilirubin encephalopathy
BIND, bilirubin-induced neurological disorders
AUC, area under curve
NPV, negative predictive value
PPV, positive predictive value
ROC, receiver operating characteristic
SHB, significant hyperbilirubinemia
TcB, transcutaneous bilirubin
TSB, total serum bilirubin
95% CI, 95% confidence interval
