# Supplementary material for: The use of transcutaneous bilirubin nomograms for the prevention of bilirubin neurotoxicity in the neonates
Source: Front Public Health. 2023 Jul 19;11:1212667. doi: 10.3389/fpubh.2023.1212667 (PMC10395091; doi:10.3389/fpubh.2023.1212667)
Supplement: Supplementary file 1 [file Table_1.docx]

| Forehead | | Nomograms without subsequent phototherapy | | | | | | |  |
| --- | --- | --- | --- | --- | --- | --- | --- | --- | --- |
|  |  | **5th percentile** | **10th percentile** | **25th percentile** | **50th percentile** | **75th percentile** | **90th percentile** | **95th percentile** | **50th percentile with subsequent phototherapy** |
|  | 1 - 12 hrs | 0.150 | 0.172 | 0.208 | 0.237 | 0.265 | 0.293 | 0.329 | **0.334** |
|  | 13 - 24 hrs | 0.093 | 0.119 | 0.149 | 0.177 | 0.195 | 0.216 | 0.234 | **0.265** |
|  | 25 - 36 hrs | 0.046 | 0.075 | 0.101 | 0.127 | 0.140 | 0.155 | 0.159 | **0.202** |
|  | 37 - 48 hrs | 0.010 | 0.040 | 0.064 | 0.086 | 0.097 | 0.108 | 0.105 | **0.147** |
|  | 49 - 60 hrs | -0.016 | 0.013 | 0.036 | 0.055 | 0.067 | 0.077 | 0.071 | **0.094** |
|  | 61 - 72 hrs | -0.031 | -0.004 | 0.020 | 0.034 | 0.050 | 0.060 | 0.058 | **0.049** |
|  | 73 - 84 hrs | -0.036 | -0.012 | 0.014 | 0.023 | 0.047 | 0.059 | 0.063 |  |
|  | 85 - 96 hrs | -0.031 | -0.012 | 0.018 | 0.021 | 0.058 | 0.073 | 0.090 |  |
| Sternum | |  |  |  |  |  |  |  |  |
|  | 1 - 12 hrs | 0.157 | 0.185 | 0.211 | 0.237 | 0.262 | 0.282 | 0.300 | **0.322** |
|  | 13 - 24 hrs | 0.093 | 0.123 | 0.157 | 0.183 | 0.203 | 0.224 | 0.241 | **0.263** |
|  | 25 - 36 hrs | 0.045 | 0.073 | 0.109 | 0.137 | 0.154 | 0.174 | 0.187 | **0.207** |
|  | 37 - 48 hrs | 0.013 | 0.034 | 0.071 | 0.097 | 0.114 | 0.132 | 0.140 | **0.157** |
|  | 49 - 60 hrs | -0.004 | 0.008 | 0.040 | 0.064 | 0.083 | 0.096 | 0.097 | **0.109** |
|  | 61 - 72 hrs | -0.004 | -0.006 | 0.018 | 0.039 | 0.062 | 0.069 | 0.059 | **0.065** |
|  | 73 - 84 hrs | 0.011 | -0.008 | 0.006 | 0.022 | 0.049 | 0.048 | 0.027 |  |
|  | 85 - 96 hrs | 0.041 | 0.002 | 0.002 | 0.012 | 0.046 | 0.036 | 0.000 |  |

Supplementary Table 1: Rate of increase of transcutaneous bilirubin (TcB) for nomogram percentile [mg/dL per hrs]
