# Supplementary material for: The use of transcutaneous bilirubin nomograms for the prevention of bilirubin neurotoxicity in the neonates
Source: Front Public Health. 2023 Jul 19;11:1212667. doi: 10.3389/fpubh.2023.1212667 (PMC10395091; doi:10.3389/fpubh.2023.1212667)
Supplement: Supplementary file 2 [file Table_2.docx]

**Supplementary Table 2** Age-specific forehead TcB levels at different percentile ranges and their predictive ability for subsequent need of phototherapy. TcB: transcutaneous bilirubin; NPV: negative predictive value; PPV: positive predictive value; SHB: significant hyperbilirubinemia

| 0-12 hrs |  |  |  |  |  |  |  |
| --- | --- | --- | --- | --- | --- | --- | --- |
| Percentile | Number (N=268) | SHB+ | SHB- | PPV | NPV | Sensitivity | Specificity |
| Above 95th percentile | 27 | 7 | 20 | 26% | 93% | 30% | 92% |
| Below 95th percentile | 241 | 16 | 225 |  |  |  |  |
| Above 75th percentile | 99 | 11 | 88 | 11% | 93% | 48% | 64% |
| Below 75th percentile | 169 | 12 | 157 |  |  |  |  |
| Above 40th percentile | 203 | 17 | 186 | 8% | 91% | 74% | 24% |
| Below 40th percentile | 65 | 6 | 59 |  |  |  |  |
| 12- 24 hrs |  |  |  |  |  |  |  |
| Percentile | Number (N=288) | SHB+ | SHB- | PPV | NPV | Sensitivity | Specificity |
| Above 95th percentile | 27 | 9 | 18 | 33% | 94% | 36% | 93% |
| Below 95th percentile | 261 | 16 | 245 |  |  |  |  |
| Above 75th percentile | 110 | 20 | 90 | 18% | 97% | 80% | 66% |
| Below 75th percentile | 178 | 5 | 173 |  |  |  |  |
| Above 40th percentile | 197 | 23 | 174 | 12% | 98% | 92% | 34% |
| Below 40th percentile | 91 | 2 | 89 |  |  |  |  |
| 25-36 hrs |  |  |  |  |  |  |  |
| Percentile | Number (N=288) | SHB+ | SHB- | PPV | NPV | Sensitivity | Specificity |
| Above 95th percentile | 24 | 7 | 17 | 29% | 94% | 29% | 94% |
| Below 95th percentile | 264 | 17 | 247 |  |  |  |  |
| Above 75th percentile | 93 | 19 | 74 | 20% | 97% | 79% | 72% |
| Below 75th percentile | 195 | 5 | 190 |  |  |  |  |
| Above 40th percentile | 196 | 23 | 173 | 12% | 99% | 96% | 34% |
| Below 40th percentile | 92 | 1 | 91 |  |  |  |  |
| 37-48 hrs |  |  |  |  |  |  |  |
| Percentile | Number (N=286) | SHB+ | SHB- | PPV | NPV | Sensitivity | Specificity |
| Above 95th percentile | 26 | 10 | 16 | 38% | 97% | 53% | 94% |
| Below 95th percentile | 260 | 9 | 251 |  |  |  |  |
| Above 75th percentile | 93 | 17 | 76 | 18% | 99% | 89% | 72% |
| Below 75th percentile | 193 | 2 | 191 |  |  |  |  |
| Above 40th percentile | 189 | 18 | 171 | 10% | 99% | 95% | 36% |
| Below 40th percentile | 97 | 1 | 96 |  |  |  |  |
| 49-60 hrs |  |  |  |  |  |  |  |
| Percentile | Number (N=278) | SHB+ | SHB- | PPV | NPV | Sensitivity | Specificity |
| Above 95th percentile | 21 | 5 | 16 | 24% | 97% | 42% | 94% |
| Below 95th percentile | 257 | 7 | 250 |  |  |  |  |
| Above 75th percentile | 88 | 10 | 78 | 11% | 99% | 83% | 71% |
| Below 75th percentile | 190 | 2 | 188 |  |  |  |  |
| Above 40th percentile | 181 | 12 | 169 | 7% | 100% | 100% | 36% |
| Below 40th percentile | 97 | 0 | 97 |  |  |  |  |
| 61-72 hrs |  |  |  |  |  |  |  |
| Percentile | Number (N=273) | SHB+ | SHB- | PPV | NPV | Sensitivity | Specificity |
| Above 95th percentile | 22 | 6 | 16 | 27% | 99% | 67% | 94% |
| Below 95th percentile | 251 | 3 | 248 |  |  |  |  |
| Above 75th percentile | 82 | 9 | 73 | 11% | 100% | 100% | 72% |
| Below 75th percentile | 191 | 0 | 191 |  |  |  |  |
| Above 40th percentile | 173 | 9 | 164 | 5% | 100% | 100% | 38% |
| Below 40th percentile | 100 | 0 | 100 |  |  |  |  |
