# Supplementary material for: The use of transcutaneous bilirubin nomograms for the prevention of bilirubin neurotoxicity in the neonates
Source: Front Public Health. 2023 Jul 19;11:1212667. doi: 10.3389/fpubh.2023.1212667 (PMC10395091; doi:10.3389/fpubh.2023.1212667)
Supplement: Supplementary file 3 [file Table_3.docx]

**Supplementary Table 3** Age-specific sternum TcB levels at different percentile ranges and their predictive ability for subsequent need of phototherapy.TcB: transcutaneous bilirubin; NPV: negative predictive value; PPV: positive predictive value; SHB: significant hyperbilirubinemia

| 1- 12 hrs |  |  |  |  |  |  |  |
| --- | --- | --- | --- | --- | --- | --- | --- |
| Percentile | Number (N=268) | SHB+ | SHB- | PPV | NPV | Sensitivity | Specificity |
| Above 95th percentile | 31 | 8 | 23 | 26% | 94% | 35% | 91% |
| Below 95th percentile | 237 | 15 | 222 |  |  |  |  |
| Above 75th percentile | 105 | 17 | 88 | 16% | 96% | 74% | 64% |
| Below 75th percentile | 163 | 6 | 157 |  |  |  |  |
| Above 40th percentile | 201 | 23 | 178 | 11% | 100% | 100% | 27% |
| Below 40th percentile | 67 | 0 | 67 |  |  |  |  |
| 12- 24 hrs |  |  |  |  |  |  |  |
| Percentile | Number (N=288) | SHB+ | SHB- | PPV | NPV | Sensitivity | Specificity |
| Above 95th percentile | 31 | 11 | 20 | 35% | 95% | 44% | 92% |
| Below 95th percentile | 257 | 14 | 243 |  |  |  |  |
| Above 75th percentile | 105 | 19 | 86 | 18% | 97% | 76% | 67% |
| Below 75th percentile | 183 | 6 | 177 |  |  |  |  |
| Above 40th percentile | 200 | 25 | 175 | 13% | 100% | 100% | 33% |
| Below 40th percentile | 88 | 0 | 88 |  |  |  |  |
| 25-36 hrs |  |  |  |  |  |  |  |
| Percentile | Number (N=287) | SHB+ | SHB- | PPV | NPV | Sensitivity | Specificity |
| Above 95th percentile | 23 | 9 | 14 | 39% | 94% | 38% | 95% |
| Below 95th percentile | 264 | 15 | 249 |  |  |  |  |
| Above 75th percentile | 96 | 22 | 74 | 23% | 99% | 92% | 72% |
| Below 75th percentile | 191 | 2 | 189 |  |  |  |  |
| Above 40th percentile | 197 | 24 | 173 | 12% | 100% | 100% | 34% |
| Below 40th percentile | 90 | 0 | 90 |  |  |  |  |
| 37-48 hrs |  |  |  |  |  |  |  |
| Percentile | Number (N=286) | SHB+ | SHB- | PPV | NPV | Sensitivity | Specificity |
| Above 95th percentile | 28 | 11 | 17 | 39% | 97% | 58% | 94% |
| Below 95th percentile | 258 | 8 | 250 |  |  |  |  |
| Above 75th percentile | 100 | 18 | 82 | 18% | 99% | 95% | 69% |
| Below 75th percentile | 186 | 1 | 185 |  |  |  |  |
| Above 40th percentile | 193 | 19 | 174 | 10% | 100% | 100% | 35% |
| Below 40th percentile | 93 | 0 | 93 |  |  |  |  |
| 49-60 hrs |  |  |  |  |  |  |  |
| Percentile | Number (N=277) | SHB+ | SHB- | PPV | NPV | Sensitivity | Specificity |
| Above 95th percentile | 28 | 8 | 20 | 29% | 98% | 67% | 92% |
| Below 95th percentile | 249 | 4 | 245 |  |  |  |  |
| Above 75th percentile | 90 | 12 | 78 | 13% | 100% | 100% | 71% |
| Below 75th percentile | 187 | 0 | 187 |  |  |  |  |
| Above 40th percentile | 180 | 12 | 168 | 7% | 100% | 100% | 37% |
| Below 40th percentile | 97 | 0 | 97 |  |  |  |  |
| 61-72 hrs |  |  |  |  |  |  |  |
| Percentile | Number (N=273) | SHB+ | SHB- | PPV | NPV | Sensitivity | Specificity |
| Above 95th percentile | 21 | 6 | 15 | 29% | 99% | 67% | 94% |
| Below 95th percentile | 252 | 3 | 249 |  |  |  |  |
| Above 75th percentile | 80 | 9 | 71 | 11% | 100% | 100% | 73% |
| Below 75th percentile | 193 | 0 | 193 |  |  |  |  |
| Above 40th percentile | 173 | 9 | 164 | 5% | 100% | 100% | 38% |
| Below 40th percentile | 100 | 0 | 100 |  |  |  |  |
