# Supplementary material for: The use of transcutaneous bilirubin nomograms for the prevention of bilirubin neurotoxicity in the neonates
Source: Front Public Health. 2023 Jul 19;11:1212667. doi: 10.3389/fpubh.2023.1212667 (PMC10395091; doi:10.3389/fpubh.2023.1212667)
Supplement: Supplementary file 4 [file Data_Sheet_1.docx]

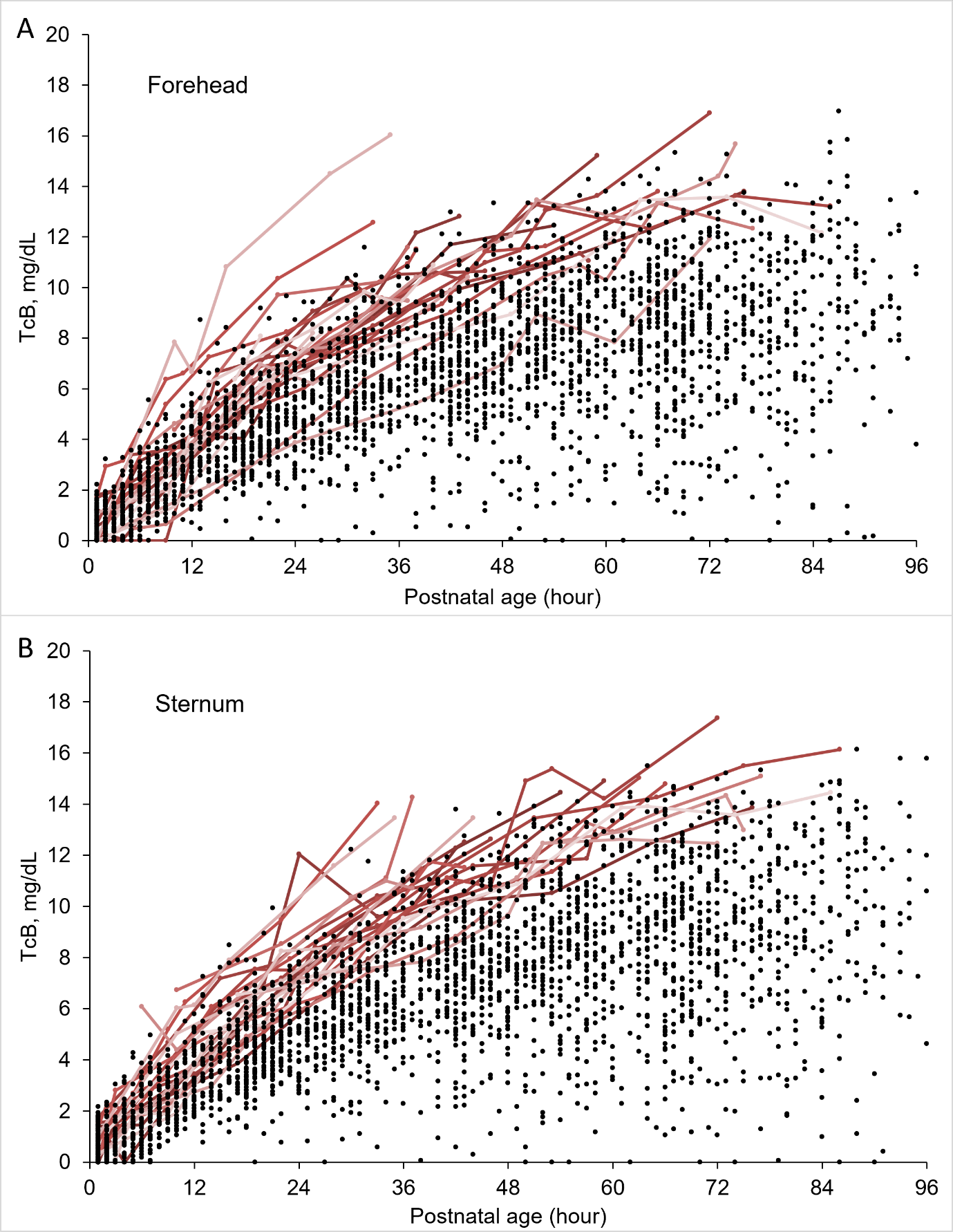


Supplementary Figure 1: Transcutaneous bilirubin (TcB) individual measurements (mg/dL) performed on the: (A) forehead and (B) sternum. The x-axis shows postnatal age in hours. Red lines represent measurements taken from individual neonates who required subsequent phototherapy.
